# Extracellular Microvesicles (MV’s) Isolated from 5-Azacytidine-and-Resveratrol-Treated Cells Improve Viability and Ameliorate Endoplasmic Reticulum Stress in Metabolic Syndrome Derived Mesenchymal Stem Cells

**DOI:** 10.1007/s12015-020-10035-4

**Published:** 2020-09-03

**Authors:** C Weiss, K Kornicka-Grabowska, M Mularczyk, N Siwinska, K Marycz

**Affiliations:** 1 PferdePraxis Dr. Med. Vet. Daniel Weiss, Postmatte 14, CH-8807 Freienbach, Switzerland; 2 Department of Experimental Biology, Wroclaw University of Environmental and Life Sciences, Norwida 27B street, A7 building, 50-375 Wroclaw, Poland; 3 International Institute of Translational Medicine, Malin, Jesionowa 11, 55-114 Wisznia Mała, Poland; 4 Department of Internal Medicine and Clinic of Diseases of Horses, Dogs and Cats, Faculty of Veterinary Medicine, Wroclaw University of Environmental and Life Sciences, Pl. Grunwaldzki 47, 50-366 Wroclaw, Poland; 5 Faculty of Veterinary Medicine, Equine Clinic-Equine Surgery, Justus-Liebig-University, 35392 Giessen, Germany

**Keywords:** Stem cells, Extracellular vesicles, Rejuvenation

## Abstract

Extracellular vesicles (EVs), a spherical membrane fragments including exosomes, are released from several cell types, including mesenchymal stromal cells (MSCs), constitutively or under stimulation. As MVs cargo include DNA, RNA, miRNA, lipids and proteins their have gain special attention in the field of regenerative medicine. Depending on the type of transferred molecules, MVs may exert wide range of biological effects in recipient cells including pro-inflammatory and anti-apoptotic action. In presented paper, we isolated MVs form adipose derived mesenchymal stem cells (ASC) which underwent stimulation with 5-azacytydine and resveratrol (AZA/RES) in order to improve their therapeutic potential. Then, isolated MVs were applied to ASC with impaired cytophysiological properties, isolated from equine metabolic syndrome diagnosed animals. Using RT-PCR, immunofluorescence, ELISA, confocal microscopy and western blot, we have evaluated the effects of MVs on recipient cells. We have found, that MVs derived from AZA/RES treated ASC ameliorates apoptosis, senescence and endoplasmic reticulum (ER) stress in deteriorated cells, restoring their proper functions. The work indicates, that cells treated with AZA/RES through their paracrine action can rejuvenate recipient cells. However, further research needs to be performed in order to fully understand the molecular mechanisms of these bioactive factors action.

**Electronic supplementary material:**

The online version of this article (10.1007/s12015-020-10035-4) contains supplementary material, which is available to authorized users.

**Graphical Abstract:**
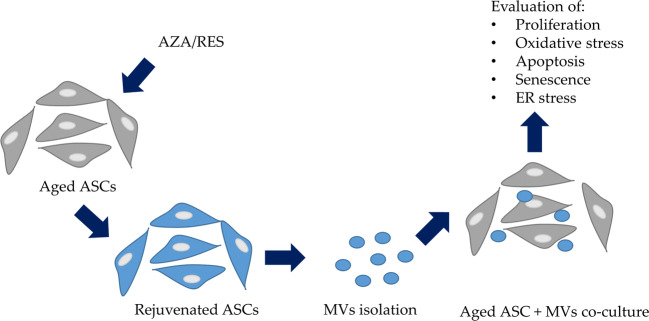
Graphical abstract of presented study

## Introduction

Regenerative medicine therapies based on the application of stem cells hold grate promise not only for the treatment of musculoskeletal system disorders but also in the course of endocrine diseases [1, 2]. Sedentary life style, obesity, insulin resistance or insulin dysregulations are common features associated with type II diabetes (T2D) or syndrome X (MetS) development. Recently, due to increased prevalence, MetS have become a subject of extensive research in human as well as in veterinary endocrinology. Equine metabolic syndrome (EMS) is characterized by insulin resistance, past or chronic laminitis and adiposity in specific location such as around eyes or on the base of the tail [3]. Nowadays, EMS is frequently diagnosed disease affecting horses population worldwide and if not treated properly, may lead to the development of laminitis – a life-threatening disease [3]. Interestingly, laminitis can be partially compared to cardiovascular complications occurring in humans during metabolic disorders. Furthermore, more and more research have proven that horse model have been applied for the study of certain diseases in humans thus possess great potential for translational medicine [4–6]. Therefore, an equine model of metabolic syndrome is proposed for translational research in humans regarding metabolic disorders and their consequences.

Recently, more and more attention has been paid toward application of stem cells for treatment of endocrine disorders including T2D or EMS. Due to their unique properties, abundance and ease of isolation, mesenchymal progenitor cells (MSC) from bone marrow (BMSC) and adipose tissue (ASC) are under intensive investigation in multiple clinical trials all over the world [7–11]. MSCs are characterized by multilineage differentiation potential, anti-inflammatory as well as immunomodulatory properties which are responsible for therapeutic potential of MSC in the course of different disorders including T2D and EMS [1, 12–16]. As recently showed, the plausible mechanism of MSC action can be at least partially explain by their paracrine activity. MSCs secrete extracellular microvesicles (MVs)- a spherical membrane fragments including exosomes which carry different type of biological cargo, including proteins, peptides, mRNA, lipids and miRNA [17]. For that reason, MVs similar to the cells of origin, are characterised by great therapeutic potential and since now have been successfully applied in the treatment of multiple disorders including liver, kidney, lung myocardial injuries or EMS [18–20]. Numerous studies showed, that MV’s are rich in growth factors which induce and mediate regeneration process e.g. vascular endothelial growth factor (VEGF), insulin-like growth factor 1 (IGF-1), basic fibroblast growth factors (bFGF), interleukin 6 (IL-6), chemokine (C-C motif) ligand 2 (CCL-2) and hepatocyte growth factor (HGF) [17, 21–23]. It was shown, that MVs are able to modulate immune response, diminish inflammation and modulate regenerative properties of recipient cells [17, 23]. However, the pro-regenerative properties of MV’s strongly depends on the physiological condition of MSCs from which they originate. In our previous research, we demonstrated, that EMS derived ASC suffer from reduced proliferative activity, enhanced apoptosis and abundant oxidative stress factors accumulation which leads to their senescence [24, 25]. Moreover, it was showed, that insulin resistance impairs multilineage differentiation potential of EMS derived ASCs due to amelioration of mitochondrial biogenesis and dynamics [26]. As a result of insulin resistance, impairment of autophagy and mitophagy occurs, leading to deterioration of ASCs pro-regenerative potential. In consequence application of autologous ASC during EMS is limited and may not exert expected therapeutic outcome. However, our previous studies have shown that a combination of 5-azacytydine (AZA) and resveratrol (RES) is able to reverse aged phenotype of these cells. It was shown, that AZA/RES increase proliferative potential, reduces apoptosis and improve multilineage differentiation potential of ASC derived from EMS individuals [27–29]. Rejuvenated ASCs has more abundantly produced MVs rich in immunomodulatory factors which serves as anti-oxidative defense against free radicals produce under EMS condition and modulate activity of immune cells [29]. Therefore, in presented study we decided to further investigate the biological activity of MVs isolated from AZA/RES treated cells. It was investigated, whether similar to AZA/RES, MVs are originating from rejuvenated cells can modulate apoptosis, oxidative stress and mitophagy in recipient progenitor cells isolated from EMS horses. As it was demonstrated, that the combination of AZA/RES abolish negative consequences of free radicals accumulation and rejuvenate impaired cells, we hypothesized that MVs originating from these cells are also able to improve cytophysiological properties of recipient cells.

## Results

### Evaluation of Cellular Viability

**Fig. 1:**
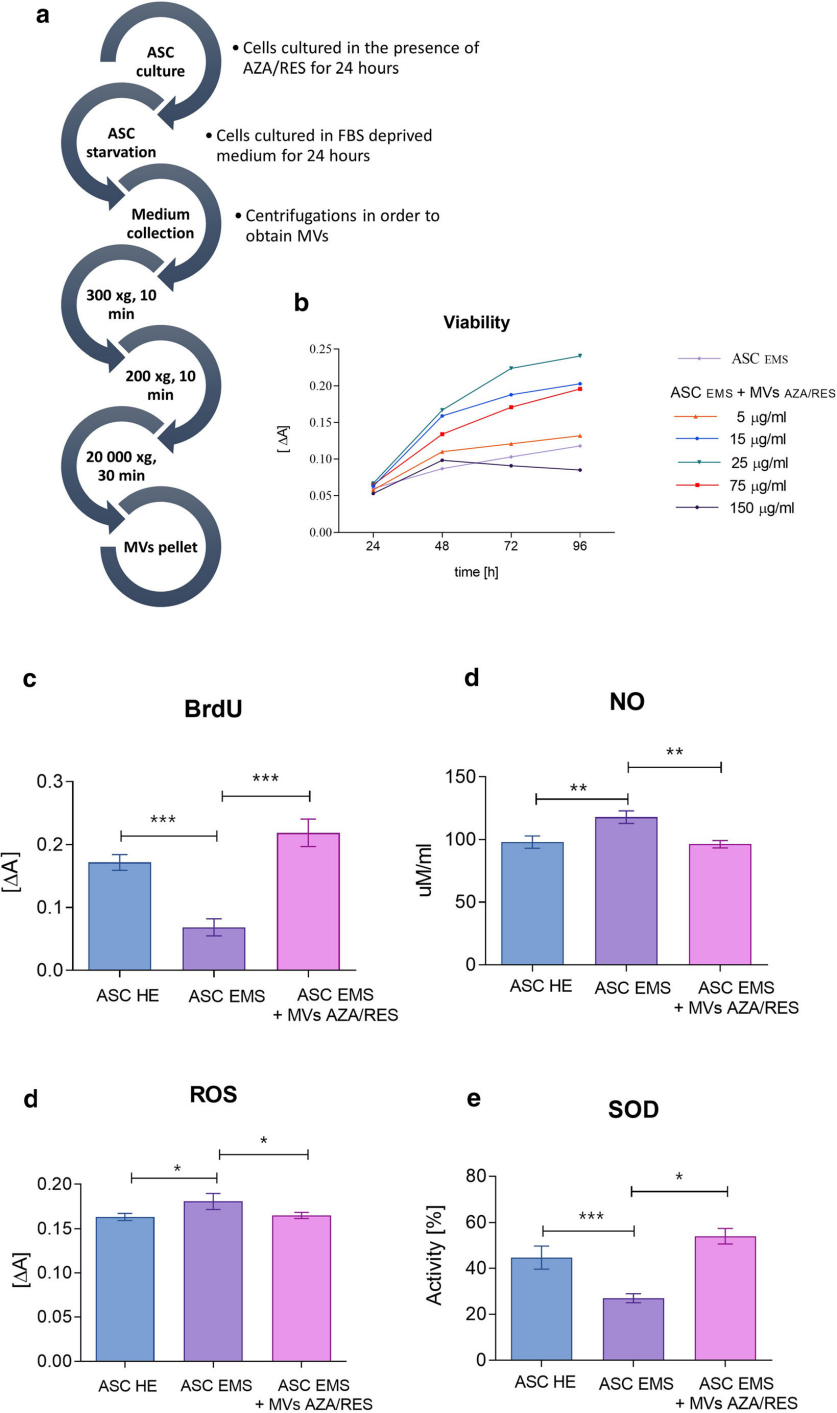
Evaluation of viability and oxidative stress factors in cells. Scheme of MVs isolation (**a**). The influence of different MVs concentration on cells (**b**). Levels of BrdU incorporation (**c**), NO (**d**), ROS (**e**) and SOD (**f**) in ASC HE, ASC EMS and ASC EMS treated with 25 μg/ml MVs AZA/RES. Results expressed as mean ± SD. *P < 0.05; **P < 0.01; ***P < 0.001. **b** and **c** are reproduced from Kornicka-Garbowska et al. under Creative Commons licence (https://www.ncbi.nlm.nih.gov/pmc/articles/PMC6921487/)

Scheme of MVs isolation is shown at Fig. 1a. In order to select most beneficial concentration, Alamar blue assay was performed (Fig. 1b). Cells were cultured for 24 h with five different concentration of MVs and their viability was established after 24, 48, 72 and 96 h of culture. MVs concentration equalled 25 μg/ml was shown to significantly enhance cellular viability. For that reason,it was selected and applied in further experiments. Obtained results indicated on decreased proliferation in ASC EMS however it was enhanced after treatment of cells with MVs AZA/RES (Fig. 1c). Treatment of cells with MVs AZA/RES reduced amount of NO (Fig. 1d) and ROS (Fig. 1e) while increased activity of SOD (Fig. 1f).

### Assessment of Apoptosis

**Fig. 2:**
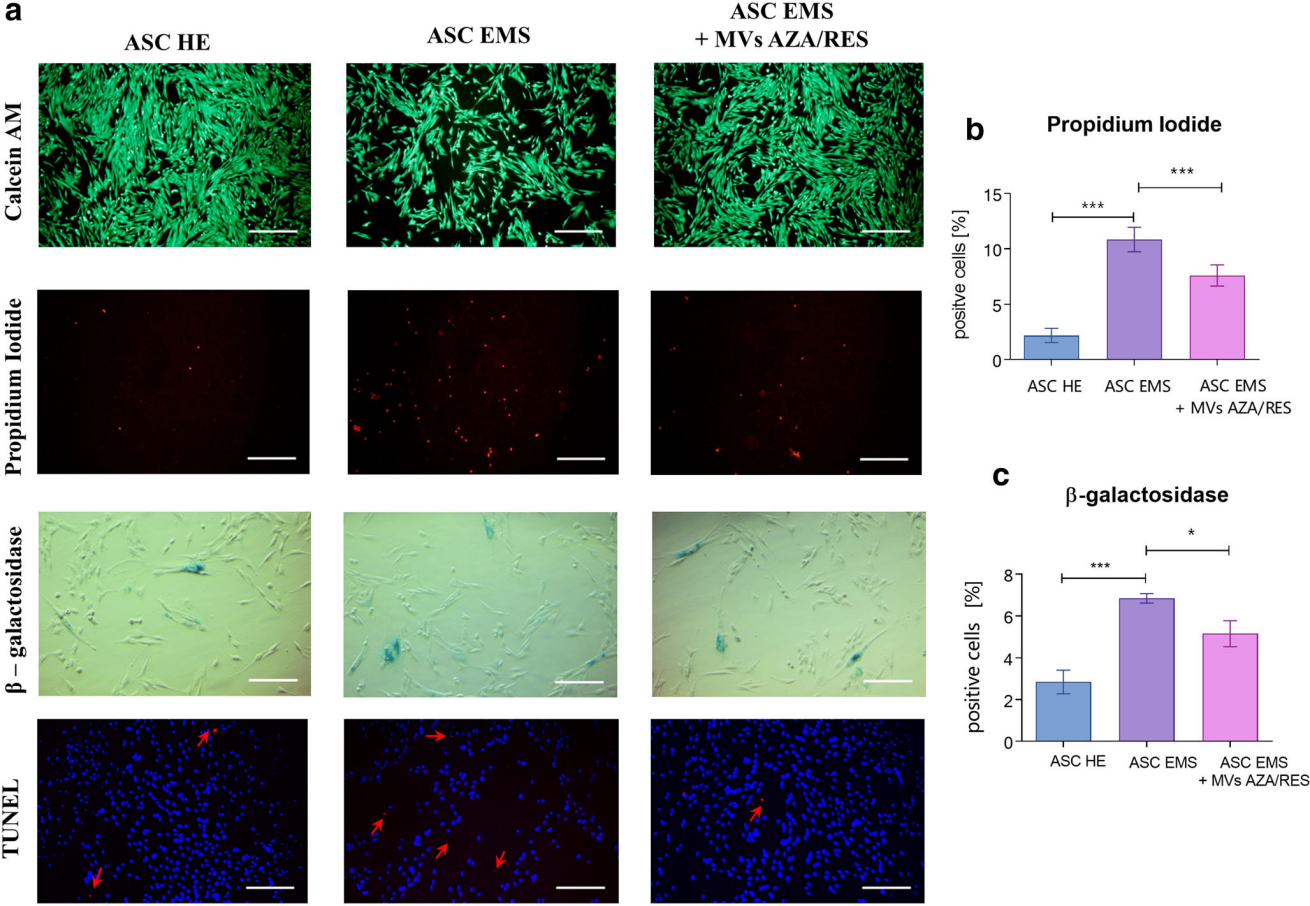

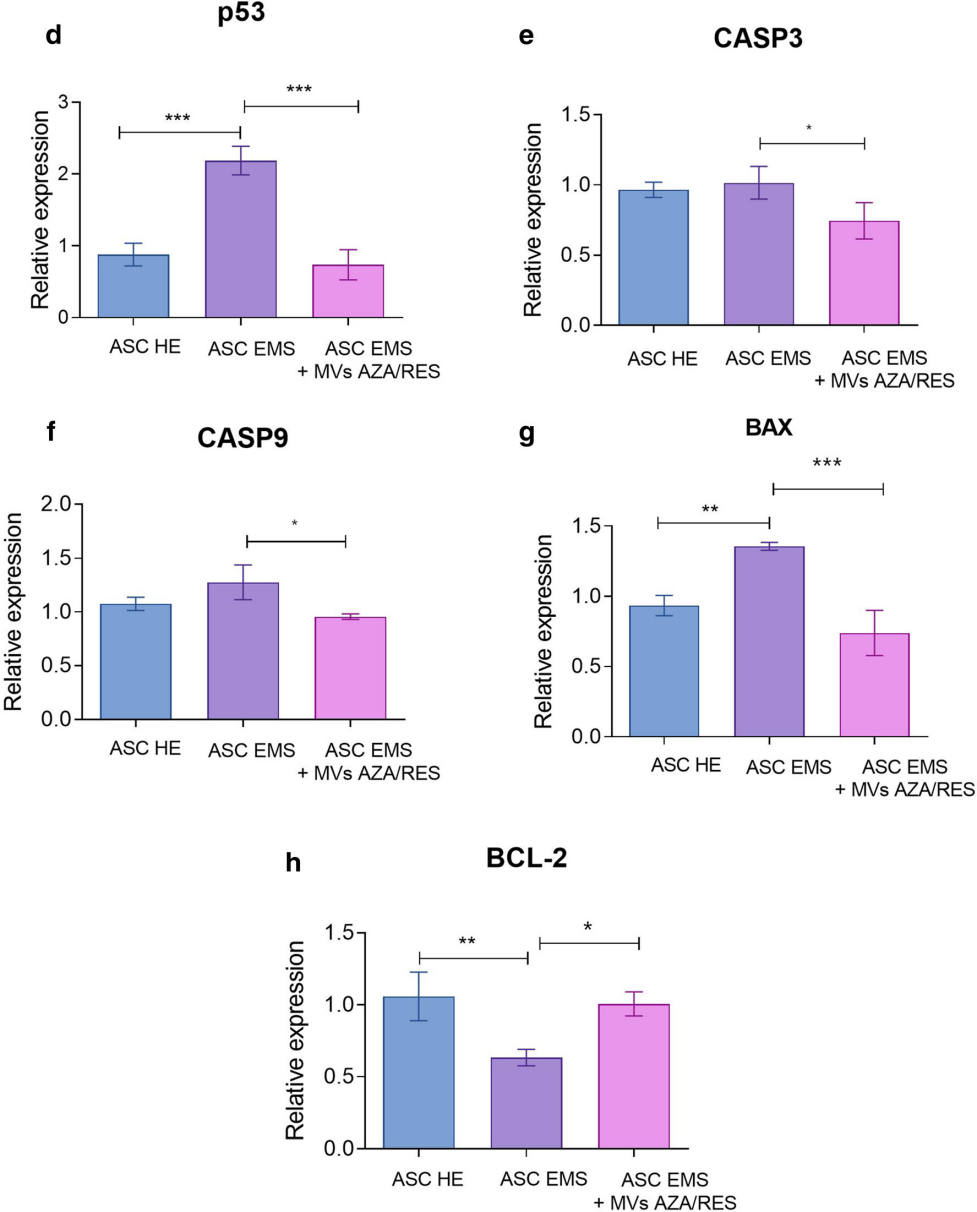
MVs from AZA/RES treated cells decreased apoptosis and senescence. Stainings for viable (Calcein) and dead cells (Propidium Iodide, TUNEL) (**a**). Percentage of dead (**b**) and senescent cells (**c**) in cultures. Expression of proapoptotic genes: p53 (**d**), CASP3 (**e**), CASP9 (**f**), BAX (**g**) and anti-apoptotic BCL-2 (**h**). Results expressed as mean ± SD. *P < 0.05; **P < 0.01; ***P < 0.001. **a**- TUNEL **d**, **g** and **h** are reproduced from Kornicka-Garbowska et al. under Creative Commons licence (https://www.ncbi.nlm.nih.gov/pmc/articles/PMC6921487/)

Live, dead cells and those accumulating β-galactosidase was visualized using specific stainings (Fig. 2a). Apoptosis was also investigated using TUNEL staining which indicated on increased number of dead cells in ASC EMS however that phenomenon was reversed after treatment of cells with MVs AZA/RES (Fig. 1a). Propidium Iodide staining (Fig. 2b) and β-galactosidase (Fig. 2c) was further quantified and presented as a percentage of positive cells. Obtained results shown that treatment of cells with MVs AZA/RES reduce number of dead and senescent cells. Expression of p53 was significantly upregulated in ASC EMS however, MVs application significantly decreased its expression (Fig. 2d). MVs treatment significantly diminished expression of caspase-3 (CASP3, Fig. 2e), caspase-9 (CASP9, Fig. 2f) and BCL2 associated X protein (BAX, Fig, 2G) in ASC EMS. Expression of BCL2 apoptosis regulator (BCL-2) was decreased in ASC EMS while comparing to control group, however MVs treatment significantly enhanced its expression (Fig. 2h).

### Evaluation of ER Stress

**Fig. 3:**
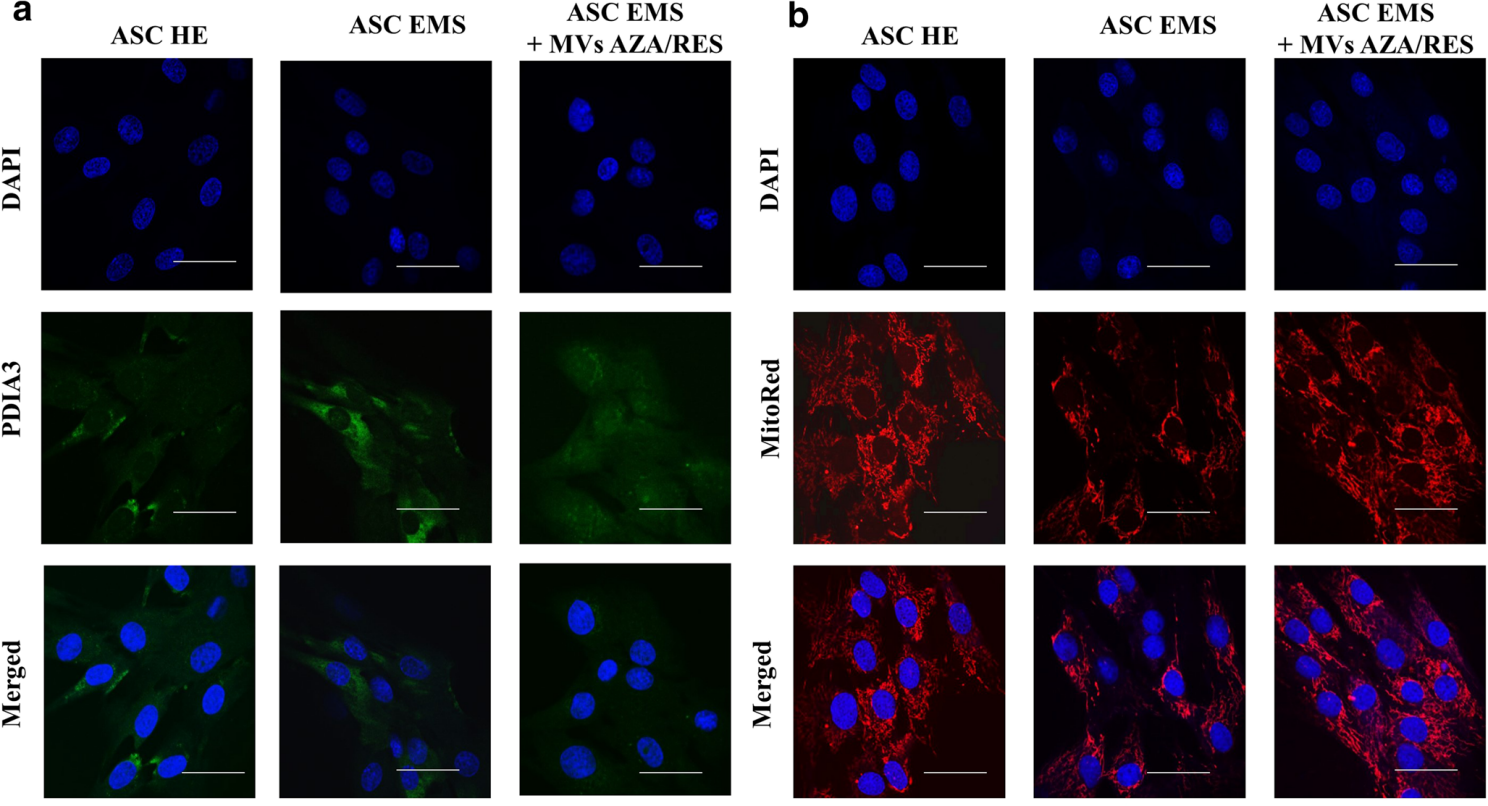

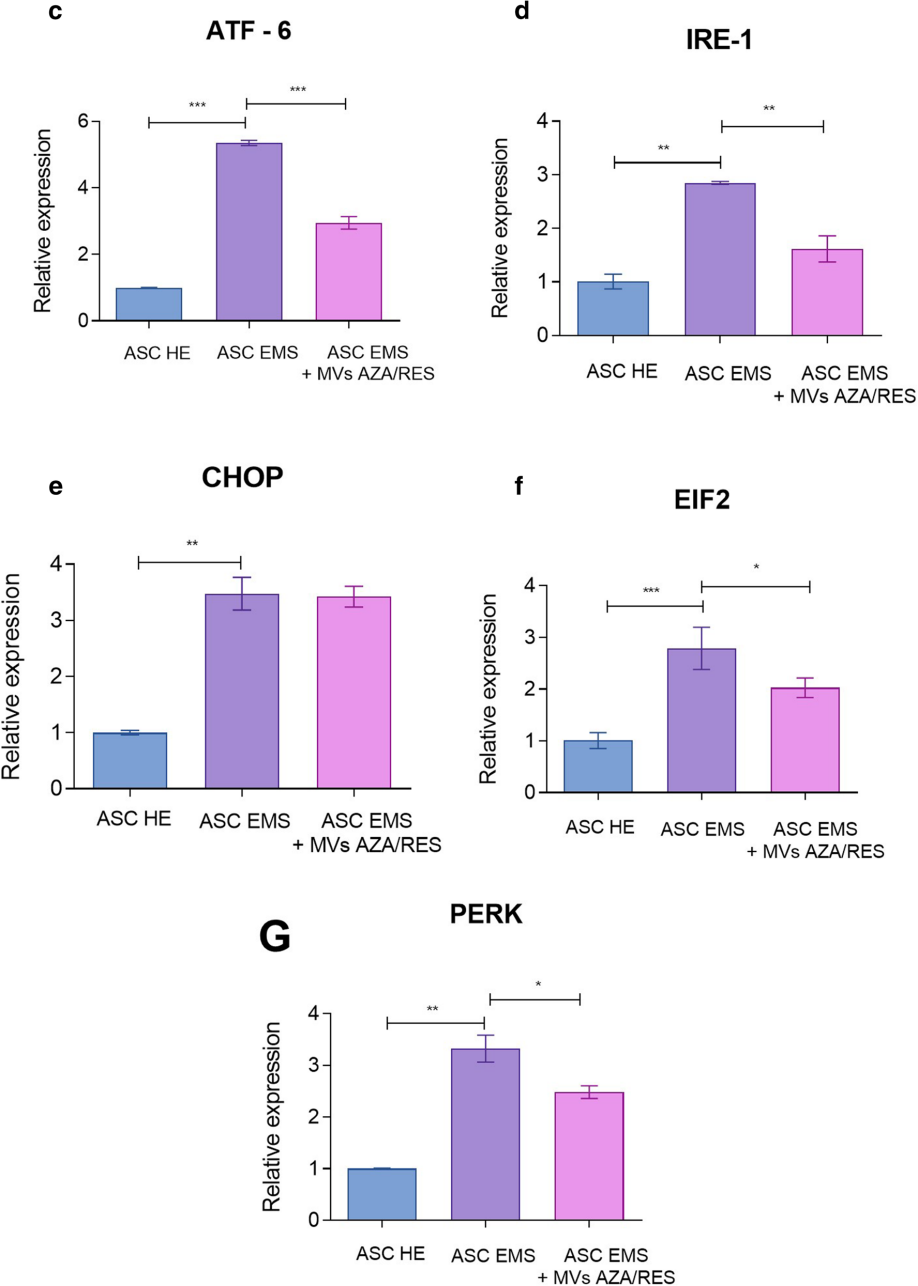
MVs from AZA/RES treated cells decreased ER stress in metabolic syndrome cells. Immunofluorescent staining for PDIA3 (**a**) and mitochondrial net visualised with MitoRed dye (**b**). Expression of genes related to ER stress response: ATF-6 (**c**), IRE-1 (**d**), CHOP (**e**), EIF2 (**f**) and PERK (**g**). Results expressed as mean ± SD. *P < 0.05; **P < 0.01; ***P < 0.001

Protein disulphide-isomerase A3 (PDIA3) was visualized in cells using immunofluorescence (Fig. 3a). Its levels was increased in ASC EMS and diminished in cells after MVs treatment. MitoRed staining (Fig. 3b) revealed decreased number of mitochondria in ASC EMS however treatment of cells with MVs restored number of mitochondria in cells. Interestingly, MVs treatment decreased expression of genes that were upregulated in ASC EMS, including Activating transcription factor 6 (ATF-6, Fig. 3c) and Inositol-Requiring kinase 1 (IRE-1, Fig.3d). No differences were observed in the expression of C/EBP Homologous Protein (CHOP, Fig. 3e) before and after MVs application. Administration of MVs decreased expression of Eukaryotic Initiation Factor 2 (EIF2, Fig. 3f) and double-stranded RNA-activated protein kinase-like ER kinase (PERK, Fig. 3g).

### Autophagy and Inflammation

**Fig. 4:**
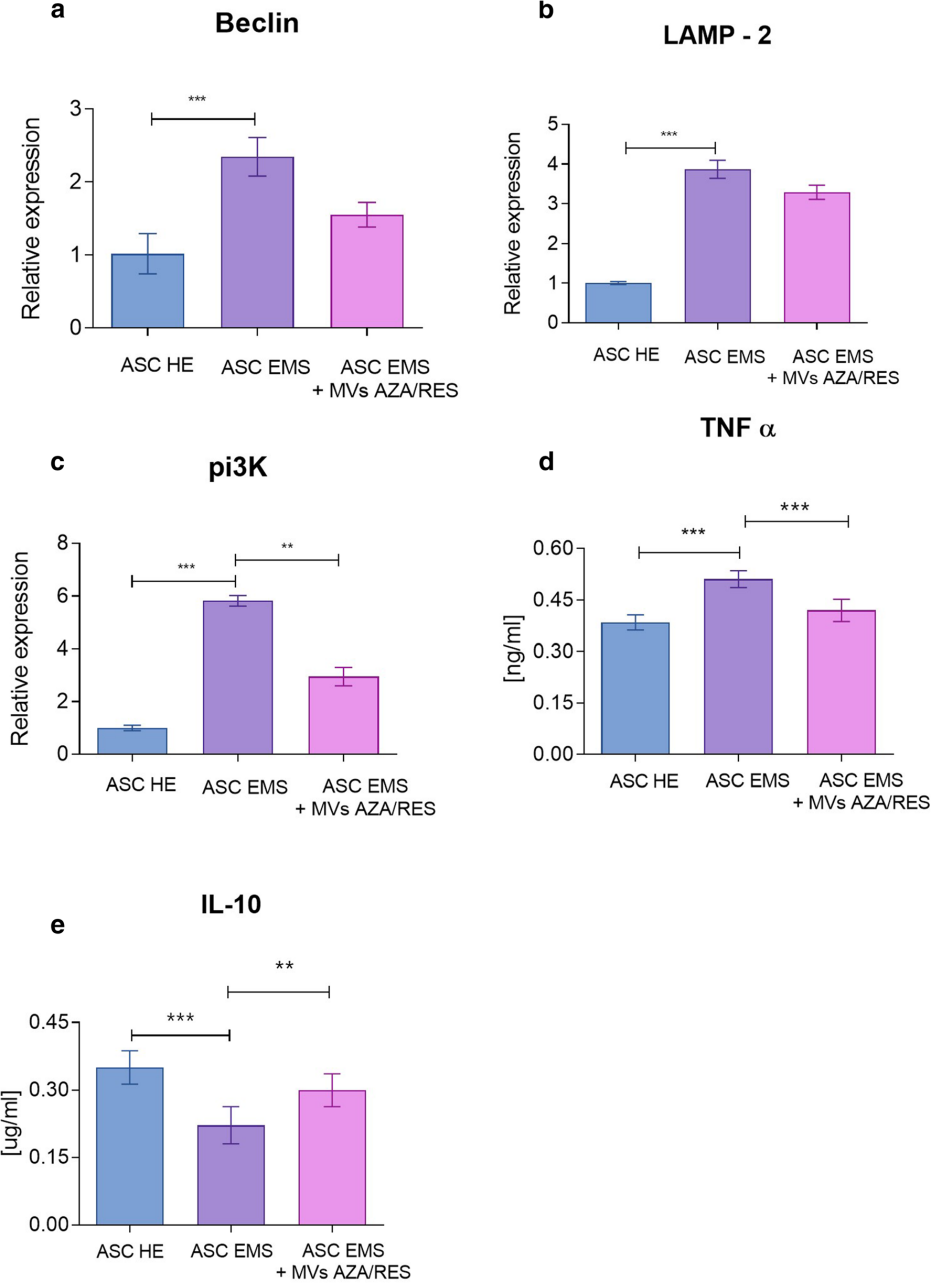
MVs from AZA/RES decreased autophagy and enhanced secretion of anti-inflammatory cytokines in treated cells. Expression of Beclin (**a**), LAMP-2 (**b**) and pi3K (**c**). Amounts of extracellular TNF α (**d**) and IL-10 (**e**) established with ELISA. Western blot for IL-6 (**f**). Results expressed as mean ± SD. *P < 0.05; **P < 0.01; ***P < 0.001

ASC EMS were characterised by increased expression of beclin (Fig. 4a) and Lysosome-associated membrane protein 2 (LAMP-2, Fig. 4b), however application of MVs did not influenced their expression. Significant increase of Phosphoinositide 3-kinase (pi3K, Fig. 4c) expression was observed in ASC EMS and application of MVs decreased its mRNA levels. ELISA results revealed increased levels of extracellular tumor necrosis factor α (TNFα, Fig. 4d) in ASC EMS however MVs application reduced its levels. ELISA for interleukin 10 (IL-10, Fig. 4e) revealed that MVs treatment resulted in its increased secretion by cells. Western blot for interleukin 6 revealed its increased levels in ASC EMS (Fig. 4f).

## Discussion

Due to increased prevalence of metabolic disorders in humans and domestic animals, development of novel and effective therapies have become a major goal in the field of regenerative medicine. Among other, adult stem cell therapies hold great promise in the treatment of insulin resistance and obesity-related inflammation. However, multiple research have indicated, that the donors age or health status are directly correlated with so called “pro-regenerative” potential of adult, mesenchymal stem cells [24, 31]. Our and other research findings have indicated, that T2D, MetS as well as EMS negatively affects ASCs multipotency, expansion properties or immunomodulatory effect which undermines their clinical utility [25, 32–34]. Moreover, in our previous research we demonstrated, that ASCs isolated form EMS horses are characterized by increased apoptosis and senescence together with mitochondria deterioration induced by enhanced systemic inflammation and excessive oxidative stress [25, 27]. Moreover, the paracrine activity of ASCs isolated form EMS is seriously deteriorated, which disturbs intercellular communication and therefore limits s “stemness” status of cells [25, 27, 29]. So far, several strategies has been proposed to reverse unfavourable phenotype of MSCs affected by dieses including their pre-incubation with chosen agents before its clinical application. For that purposes, growth factors, vitamins, amino acids or peptides has been proposed [35, 36]. Recently, we showed, that the combination of AZA/RES reversed aged phenotype of ASCs isolated from EMS (ASCs/EMS) horses- treatment resulted in increased proliferative potential, reduced oxidative stress and improved immunomodulatory properties [27, 29]. In the present study, we showed, that ASCs/EMS preincubated with AZA/RES produce MVs (MVs _AZA/RES_) of unique biological features. We have found, that MVs_AZA/RES_ positively affect cell viability and improve proliferative activity of ASCs/EMS. Furthermore, the TUNEL staining showed reduced number of dead cells among ASCs/EMS population. We have observed the significant upregulation of anti-apoptotic transcript BCL-2 and at the same time reduction of pro-apoptotic gene BAX on mRNA level. This supports the hypothesis, that MVs_AZA/RES_ not only inhibit apoptosis but also improve viability of physiologically impaired progenitor cells of EMS horses. Similar biological phenomenon was shown in a model of renal ischemia/reperfusion injury where MVs inhibited apoptosis and stimulated cellular proliferation [37]. On the other hand, Herrera et al. [38] demonstrated that in human and rat hepatocytes, MVs enhanced proliferation and decreased apoptosis through mRNA shuttled into recipient cells. Moreover, we have showed, that accumulation of senescence-associated β-galactosidase is reduced in cells treated with MVsAZA/RES. This stands in good agreement with Bruno et al. [39] who demonstrated, that MSC-derived MVs increases the proliferation rate of tubular epithelial cells after in vitro injury. Moreover, we have demonstrated reduced expression of CASP3 and CASP9, which are the crucial mediators of programmed cell apoptosis especially in the cells affected by hyperinsulinemia. It was demonstrated by Radziszewska et al. [40], that CAP3 knock-out mice were protected from streptozotocin-induced diabetes together with inhibition of B-cells proliferation through inhibition of p27 transcripts.

Common feature associated with obesity, hyperinsulinemia and insulin resistance in EMS horses is accumulation of oxidative stress factors which leads to excessive, systemic inflammation. In our previous research we showed, that adipose tissue as well as ASC residing within it suffer frm enhanced oxidative stress and inflammation, which impairs their biological functions [25, 41]. In this study, we showed, that MVsAZA/RES reduce secretion of pro-inflammatory TNFα while enhance secretion of anti-inflammatory IL-10. Recently, Hosseinkhani et al. [42] demonstrated immunomodulatory effect of MVs released from endothelial cells in the course of Atherosclerosis - a chronic and progressive inflammatory vascular disorder. Similar phenomenon was observed by Harrell et al. [43] who showed, that both local and systemic administration of MSC derived MVs efficiently suppress detrimental immune response in inflamed tissues and promote survival and regeneration of injured parenchymal cells. Additionally, by transfer of mRNA and miRNA for target cells, extracellular microvesicles promote cells survival and regenerative properties by reducing necrosis associated with oxidative stress [43]. As recently showed by our group, ASC/EMS displayed decreased proliferation rate, increased apoptosis and senescence together with mitochondria deterioration [27]. The impairment of mitochondrial biogenesis and dynamics is directly associated with deterioration of cellular function including multipotency and immune modulation [28, 29]. Numerous studies including ours, have shown that autophagy as well as mitophagy, serve as a protective mechanisms, allowing deteriorated ASC/EMS survive and maintain basic cellular functions under stress condition [27, 44, 45]. Here, for the first time we have shown that MVsAZA/RES reduce expression of transcripts involved in autophagy including LAMP-or Beclin-1. It might be explained by their anti-oxidative activity, since we observed elevated amount of SOD together with reduced ROS and NO – the master inducers of oxidative stress. Similar effect was noted by Harrell et al. [43] who showed, that MVs transferred to the target cells- injured hepatocytes, neurons and lung cells activate autophagy, inhibits apoptosis, necrosis and oxidative stress and therefore promote cellular survival and regeneration. Furthermore, it was showed, that excessive oxidative stress combined with lipo- and glucotoxicity significantly contribute to the development of ER stress [31, 46]. In the present study, we showed, that MVsAZA/RES reduce ER stress as we observed decreased expression of following transcripts ATF-6, IRE-1, PERK, EIF2 and CHOP. Similar effect was found by Liao et al. [47], who showed, that MVs could attenuate ER stress-induced apoptosis by activating AKT and ERK signaling. More recently, Chen et al. [48], showed that MSC derived MVs protects beta cells against hypoxia-induced apoptosis via miR-21 by alleviating ER stress and inhibiting p38 MAPK phosphorylation.

Recent findings in stem cells have shed a promising light for the application of their extracellular vesicles in the treatment of different disorders. In present study we showed, that ASCs/EMS preincubated with AZA/RES promote secretion of MVs that are able to reduce oxidative stress, inflammation and ER stress and thus protect recipient cells against apoptosis and senescence. However, further research are strongly required to understand the mechanism involved in regenerative processes and therapeutic potential of MV’s in the course of different disorders including EMS, T2D and MetS.

## Materials and Methods

All reagents and chemicals used in this research were purchased from Sigma-Aldrich (Poznań, Poland), unless indicated otherwise.

### Tissue Harvesting

Adipose tissue samples were harvested from the group of healthy horses (*n* = 15), and horses diagnosed with EMS. Animals were mixed sex and age – matched (8–12 years). Qualification of animals to selected group was performed on the basis of following parameters: body weight, body condition score, cresty neck score, existing laminitis, resting insulin levels, blood glucose levels, oral sugar test. Detailed characterisation of horses can be found in our previous paper [27].

### Cell Isolation and Characterisation

Samples of subcutaneous adipose tissue were harvested from the animal’s tail base. ASCs were isolated as descried previously [27] using enzymatic method (collagenase type I in a concentration of 1 mg/mL for 40 min at 37 °C) from healthy (ASC HE) and EMS horses (ASC EMS). Cells were cultured in Dulbecco’s Modified Eagle’s Medium (DMEM) Low Glucose supplemented with 10% of foetal bovine serum (FBS) and 1% of penicillin/streptomycin (PS) solution. Media were changed every 2–3 days. Cells were passaged after reaching 80% confluence using trypsin solution (TrypLE; Life Technologies, Carlsbad, CA, USA). Cells were characterised by the presence of CD44, CD45 and CD90 surface antigens using Becton Dickinson FACS Calibur Flow Cytometer as shown previously [27]. Additionally, osteogenic, chondrogenic and adipogenic differentiation of isolated cells was confirmed as well [27].

### Isolation of MVs

MVs were isolated with ultracentrifuge as described elsewhere [30]. Procedure scheme is shown in Fig. 1. In order to isolate MVs from ASC-EMS, cells were cultured with 0.5 μM of AZA and 0.05 μM of RES for 24 h as described previously [27]. Next, the medium was replaced with serum free culture medium supplemented with 1% of PS for an additional 24 h. After that, medium was collected and subjected to centrifugation at 300 xg for 10 min. Supernatant was collected and centrifuged at 2000 xg for 10 min. After that, supernatant was collected again and centrifuged in ultracentrifuge at 20000 xg for 30 min. The amount of MVs in obtained pellet was verified with BCA Protein Assay.

### Experimental Setting

In order to perform the experiments, cells were seeded in cells were seeded 24 – well plates at the density of 3 × 10^4^ per well. After 24 h, in the experimental group medium was replaced for culture medium supplemented with MVs derived from ASC-EMS AZA/RES treated cells at the concentration of 25 μg/ml. After 24 h of incubation cells were collected and subjected for further analysis.

### Proliferation Rate

In order to select most potent concentration of MVs, cells were cultured with 5 different MVs concentration for 24 h. Cell viability was evaluated using 10% resazurin-based dye-TOX-8 in accordance with manufacturer protocol, after 24, 48, 72 and 96 h of propagation. In order to perform the assay cells were incubated with dye in a CO_2_ incubator, 37 °C for 2 h and then supernatants absorbance was measured (Epoch, BioTek) at a wavelength of 600 nm for resazurin and 690 nm reference wavelength. Proliferation potential was established by the analysis of BrdU incorporation using BrdU Cell Proliferation ELISA Kit (Abcam) in accordance with manufacturer’s instructions. Briefly, cells were first incubated with anti-BrdU antibody and then with horseradish peroxidase (HRP)-conjugated goat anti-mouse antibody. Colorimetric reaction was induced by the conversion of the chromogenic substrate tetra-methylbenzidine (TMB) and the absorbance was measured using the spectrophotometer (Epoch, BioTek) at 450 nm and 550 nm as the length for the reference wave.

### Evaluation of Apoptosis and Senescence

Live and dead cells in cultures were visualized using Cellstain Double Staining Kit in accordance with manufacturers protocol. Viable cells were stained with Calcein-AM (green fluorescence), whereas dead cells’ nuclei were stained with Propidium Iodide (orange fluorescence). Cells were then observed using fluorescence microscopy (Zeiss, Axio Observer A.1). The percentage of dead cells was calculated.

Prior identification of senescence associated β-galactosidase (β-gal), cells were stained using a Senescence Cells Histochemical Staining Kit (Sigma Aldrich) in accordance with manufacturer’s protocol. Cells were then observed under an inverted microscope (Zeiss, Axio Observer A.1) and percentage of β-gal (stained blue) positive cells in regard to β-gal negative cells was calculated.

### Evaluation of Oxidative Stress Factors

Nitric oxide (NO) concentration was assessed using commercially available Griess reagent kit (Life Technologies). Superoxide dismutase (SOD) activity was measured using a SOD Assay kit (Sigma Aldrich). Reactive oxygen species (ROS) were estimated by incubating cells with an H2DCF-DA (Life Technologies) solution. All procedures were performed according to manufacturer’s protocols.

### Evaluation of TNF-α and IL-10

The amounts of extracellular TNF-α and IL-10 in culture media was investigated with ELISA assays- horse tumour necrosis factor (TNF superfamily, member 2) ELISA Kit (MyBioSource, San Diego, CA, USA) and horse Interleukin-10 ELISA Kit (MyBioSource, San Diego, CA, USA). All procedures were performed in accordance with manufacturers protocols.

### Visualization of Mitochondrial Net and ER

Mitochondrial network inside the cells was visualized using MitoRed staining. Briefly, dye solution (1:000) was added to culture media and cells were incubated for 30 min in CO_2_ incubator. Then specimens were fixed with 4%PFA and nuclei were counterstained with DAPI.

ER was visualised in cells using immunofluorescent staining for PDIA3. Briefly, cells were fixed 4% PFA, permeabilized with 0,5% Triton X-100 for 20 min and incubated with blocking buffer (10% Goat Serum, 0.2% Tween-20 in PBS). Next, specimens were kept in 4 °C with primary antibodies against PDIA3 (Abcam) diluted 1:1000 in PBS containing 1% Goat Serum and 0.2% Tween-20. Prior observations, secondary antibodies conjugated with atto-488 (dilution 1: 1000, Abcam) were applied and nuclei were counterstained with DAPI. Cells were observed and photographed with a confocal microscope (Observer Z1 Confocal Spinning Disc V.2 Zeiss).

### Gene Expression

Total RNA was isolated from cells using TriReagent in accordance with manufacturers protocol. RNA concentrations and quality were evaluated using nanospectrophotometer (Epoch, BioTek). Total RNA was reversely transcribed into cDNA using the First Strand cDNA Synthesis Kit was used (Thermo Fisher Scientific, USA). Gene expression was evaluated using SensiFast SYBR &Fluorescein Kit (Bioline, UK). T100 Thermal Cycler (Bio-Rad, USA) was used to carry out all the amplifications and detections. The 2 − ΔΔCT algorithm was used to calculate the value transcripts in relation to the expression of the reference gene – GAPDH. Primers are listed in supplementary file.

### Western Blotting

Cells were rinsed in ice-cold PBS and extracts were prepared in RIPA buffer supplemented with protease inhibitor (1:1000). Then lysates were subjected to SDS-PAGE and transferred to polyvinylidene difluoride (PVDF) membrane (BioRad). The following antibodies were used for immunoblotting: B-Actin (1:1000, Sigma Aldrich) and IL-6 (1:250, Abcam) at dilution for in in 5% non-fat milk in Tris/Nacl/Tween buffer (TBST). Then membranes were incubated with anti-rabbit and anti-mouse horseradish peroxidase-conjugated secondary antibodies. Reactions were developed using Western HRP Substrate (Millipore Corporation). Chemiluminescent signals were detected using ChemiDoc MP Imaging System (Bio-Rad, USA) and quantified with Image Lab Software (Bio-Rad, USA).

### Statistic

All experiments were performed at least in three replicates. Differences between experimental groups was estimated using the one-way ANOVA with Tukey’s test. Statistical analysis was conducted with GraphPad Prism Software (La Jolla, CA, USA). Differences were considered statistically significant at *p < 0.05, **p < 0.01, and ***p < 0.001**.**

## Electronic supplementary material

ESM 1 Sequences of primers used in qPCR. (DOCX 16 kb)
