# Supplementary material for: Extracellular Microvesicles (MV’s) Isolated from 5-Azacytidine-and-Resveratrol-Treated Cells Improve Viability and Ameliorate Endoplasmic Reticulum Stress in Metabolic Syndrome Derived Mesenchymal Stem Cells
Source: Stem Cell Rev Rep. 2020 Sep 3;16(6):1343–55. doi: 10.1007/s12015-020-10035-4 (PMC7667134; doi:10.1007/s12015-020-10035-4)
Supplement: Supplementary file 1 — Sequences of primers used in qPCR. (DOCX 16 kb) [file 12015_2020_10035_MOESM1_ESM.docx]

| Gene | Primers sequences 5′ → 3′ | Product length (bp) | Accession no. |
| --- | --- | --- | --- |
| Casp-3 | F: GGCAGACTTCCTGTATGCGT  R: CCATGGCTACCTTGCGGTTA | 167 | [XM_023630401.1](https://www.ncbi.nlm.nih.gov/entrez/viewer.fcgi?db=nucleotide&id=1333620984) |
| Bcl-2 | F: TTCTTTGAGTTCGGTGGGGT  R: GGGCCGTACAGTTCCACAA | 164 | [XM_001490436.4](https://www.ncbi.nlm.nih.gov/entrez/viewer.fcgi?db=nucleotide&id=1333703097) |
| P53 | F: : AGATAGCGATGGTCTGGC  R: TTGGGCAGTGCTCGCTTAGT | 381 | NM_001126118.1 |
| Casp-9 | F: CACCTTCCCAGGCTTTGTCT  R: GGCTCTGGCCTCAGTAAGTT | 224 | [XM_005607504.3](https://www.ncbi.nlm.nih.gov/entrez/viewer.fcgi?db=nucleotide&id=1333595432) |
| BAX | F: GGCACCTCTTCCCTCCTTTCT  R: CGATGCGCTTGAGACACTCG | 193 | [XM_023650076.1](https://www.ncbi.nlm.nih.gov/entrez/viewer.fcgi?db=nucleotide&id=1333555307) |
| ATF-6 | F: CAGGGTGCACTAGAACAGGG  R: AATGTGTCTCCCCTTCTGCG | 164 | XM_023640315.1 |
| IRE-1 | F: GAATCAGACGAGCACCCGAA  R: TTTCTTGCAGAGGCCGAAGT | 300 | \| XM_023652216.1 \| \| --- \| \| |
| CHOP | F: AGCCAAAATCAGAGCCGGAA  R: GGGGTCAAGAGTGGTGAAGG | 272 | [XM_001488999.4](https://www.ncbi.nlm.nih.gov/entrez/viewer.fcgi?db=nucleotide&id=1333686705) |
| PERK | F: GTGACTGCAATGGACCAGGA  R: TCACGTGCTCACGAGGATATT | 283 | [XM_023618757.1](https://www.ncbi.nlm.nih.gov/entrez/viewer.fcgi?db=nucleotide&id=1333577179) |
| EIF2 | F: AGTCTTCAGGCATTGGCTCC  R: CCGAGTGGGACATGTATCGG | 489 | [XM_001488848.6](https://www.ncbi.nlm.nih.gov/entrez/viewer.fcgi?db=nucleotide&id=1333700742) |
| LAMP-2 | F: GCACCCCTGGGAAGTTCTTA  R: ATCCAGCGAACACTCTTGGG | 147 | XM_014831347.1 |
| Pi3K | F: GACTTGCACTTGGGTGACATA  R: TAAGTTCCCGGAAAGTCCCC | 152 | XM_014855332.1 |
| Beclin | F: GATGCGTTATGCCCAGATGC  R: AACGGCAGCTCCTCTGAAAT | 233 | XM_014833759.1 |

Table 1 Sequences of primers used in qPCR.

# Casp-3 - Caspase-3, Bcl-2 - BCL2 apoptosis regulator, p53 - tumor protein p53, Casp-9 Caspase-9, BAX - BCL2 associated X protein, ATF-6 - Activating transcription factor 6, IRE-1 - Inositol-Requiring kinase 1 , CHOP - C/EBP Homologous Protein, PERK - double-stranded RNA-activated protein kinase-like ER kinase, eiF2- Eukaryotic Initiation Factor 2, LAMP-2 - Lysosome-associated membrane protein 2, pi3K - Phosphoinositide 3-kinases, Beclin – Beclin
